# Rhinovirus and COPD airway epithelium

**DOI:** 10.15761/PCCM.1000139

**Published:** 2017-09-07

**Authors:** Nicole Owuor, Nisha Nalamala, Joao Antonio Gimenes, Uma S. Sajjan

**Affiliations:** 1 Department of Thoracic Surgery and Medicine, Temple University, Philadelphia, PA, USA; 2 Department of Physiology, Temple University, Philadelphia, PA, USA

## Abstract

Chronic Obstructive Pulmonary Disease (COPD) is characterized by irreversible airflow limitation. It is a global disease and expected to be the third leading cause of death. Respiratory exacerbations are associated with increased mortality and morbidity in this patient population. Respiratory viruses were isolated from at least 30 to 50% of the infectious respiratory COPD exacerbations with rhinovirus being most commonly isolated pathogen. Although rhinovirus does not cause airway epithelial damage like influenza and other respiratory viruses, it may further impair innate immunity of airway epithelium, which is the first line of defense in the lungs. This may increase susceptibility to secondary bacterial infections leading to progression of lung disease. Currently, there arc no therapies available to treat rhinovirus infection in COPD and therefore understanding the mechanisms underlying RV pathogenesis in COPD is essential to identify molecular target to develop new therapeutic strategies. Quercetin, a plant polyphenol, which modulates innate immunity and effectively blocks viral replication may be useful in treating rhinovirus associated COPD exacerbations.

## Introduction

Chronic Obstructive Pulmonary Disease (COPD) is a progressive disease characterized by irreversible airflow limitation. Patients with COPD experience symptoms including coughing with excessive mucus production, shortness of breath, and chest tightness. These symptoms sre exacerbated by viral and/or bacterial infections and other environmental factors. At least 70% of the exacerbations are due to respiratory infections, out of which 30 to 50% are associated with viruses. Rhinoviruses (RV) are one of the most commonly associated viruses with viral related COPD exacerbations. RV causes self-limiting upper respiratory tract infection in healthy individuals. However, in COPD airways, RV infection is associated with persistent lung inflammation and airflow obstruction [1,2]. Additionally, RV also increases the risk for acquiring secondary bacterial infections [3] indicating that RV can impair lung innate immune mechanisms in patients with COPD.

## Rhinovirus

RVs are non-enveloped viruses with single stranded positive strand RNA genome. There are over 150 serotypes of RV categorized phylogenetically into species A. B. and C [4]. Majority of the RV-A and RV-B bind to intracellular adhesion molecule 1 (ICAM-1), and the remaining bind to Low Density Lipoprotein Receptor (LDLR) family members [5,6]. Human cadherin family member 3was recently identified as a receptor for RV-C [7]. RV-A and RV-B enter the cells by receptor-mediated endocytosis followed by uncoating to release the genome into the cytoplasm, where the virus replicates [1]. The entry and uncoating mechanism for RV-C is not well-known. Compared to RV-A and RV-B, RV-C is associated with severe lung disease in patients with chronic lung disease [8].

## Airway epithelium

The primary target for RV infection is airway epithelium that lines the conductive airways. RV, which was thought to cause only upper respiratory tract infections, was also detected in ciliated cells of lower airways [9]. RV infects small proportion of ciliated cells in both and upper and lower airways. However, experimental evidence indicate that the basal cells of airway epithelium are more amenable to RV infection than ciliated cells [10]. Basal cell hyperplasia and leaky airway epithelium which can potentially expose basal cells are some of the features that are found in COPD airways [11,12] and this may increase susceptibility to RV infection. Since RV does not cause significant cell death, differences in the host immune responses are thought to be responsible for variable symptoms. Airway epithelial cells responds to RV infection by producing chemokines and growth factors to recruit other innate immune cells, which in addition to contributing to local inflammation and cold symptoms may also aid viral clearance and resolution of inflammation at later stages of infection. In addition, airway epithelial cells also express type I interferons, which can induce interferon-stimulated genes that inhibit viral replication. Therefore, we believe that appropriate airway epithelial responses are crucial in determining the viral clearance and resolution of inflammation following RV infection.

Previously, we have shown that mucociliary airway epithelial cell cultures established from COPD airway progenitor cells show goblet cell hyperplasia and produce more C-X-C chemokines under unstimulated conditions compared to similarly cultured airway progenitor cells from healthy non-smokers [13,14]. Expression of CXC-chemokine, IL-8 was partially due to attenuated activation of FOX03A and persistent activation of EGF receptor in COPD airway epithelial cells [14]. Additionally, COPD airway epithelial cell cultures also show evidence of epithelial to mesenchymal transition and this is thought to be due to increased activation of signaling pathways downstream of TGF-β1 [12,15]. Furthermore, progenitor cells from COPD patients was shown to be defective in regenerating normal airway epithelium [16]. These observations indicate that airway progenitor cells in COPD may have acquired epigenetic changes due to persistent exposure to cigarette smoke and inflammatory milieu and these changes affect the regeneration of airway epithelium leading to abnormal airway epithelium.

Abnormality in airway epithelium in structure or function may significantly affect the outcome of infection. Previously, we demonstrated that compared to normal, mucociliary-differentiated COPD airway epithelial cell cultures show exaggerated chemokine responses and antiviral interferon responses [13]. Our on-going studies indicate that despite exaggerated antiviral responses COPD airway epithelial cell cultures show delay in clearing the virus and show enhanced goblet cell hyperplasia and pro-inflammatory phenotype after RV infection (Jing and Sajjan, unpublished observations). Several studies have shown similarly exaggerated chemokine responses to RV infection in cigarette smoke exposed normal airway epithelial cells indicating that cigarette smoke may be sufficient to alter the innate immune responses of these cells to infection [17,18]. Additionally RV­ infected COPD cultures also show suppressed antimicrobial factor, human β- defensin 2 to subsequent bacterial infection [19]. These findings indicate that COPD airway epithelial cells show aberrant innate immune responses to RV, and this may potentially recruit more neutrophils and other innate immune cells to the airways and increase respiratory symptoms in COPD. Consistent with this notion, experimental RV infection in mild COPD patients was accompanied by upper and lower respiratory symptoms that was more severe and prolonged compared to similarly-infected healthy non-smokers [20]. RV infection also increased oxidative and nitrosative stress markers in the lungs of COPD, but not in healthy subjects [21]. Further, this was shown to be due to reduced histone deacetylase 2 activity in macrophages. On a similar note, we showed that RV infection in mice with mild COPD phenotype resulted in persistent expression of CXCL- 1expression, recruitment of neutrophils to the airways and sustained lung inflammation [22]. Further, studies are needed to understand the mechanisms of RV-induced exaggerated responses in COPD.

## Potential therapies for rhinovirus infection

At present, there are no therapies to treat RV infection other than palliative therapies to treat symptoms of common colds. One of the reasons is that numerous serotypes of RV preclude the development of vaccines or any other specific therapies. Since RV-elaborated protease,2A, 3C plays a major role in RV replication, studies to develop treatment for RV are focused on inhibiting these proteases. Although some protease inhibitors have been shown to inhibit RV replication in vitro, their utilization as therapeutic agents is yet to be determined [23,24]. Recently, αl-antitrypsin that is expressed in the lungs and in other organs was shown to inhibit RV replication in vitro in airway epithelial cells and attenuate RV-induced neutrophilic inflammation in vivo [25]. This is quite promising since αl-antitrypsin deficient subjects develop COPD-like lung disease and are vulnerable for RV­ induced exacerbations [26]. Another potential therapy to treat RV infection is treatment with quercetin. Quercetin is a plant polyphenol with potent anti-inflammatory and antioxidant properties. We have previously demonstrated that quercetin interferes with various stages of viral life cycle, including viral entry, transcription and translation of viral genome, and processing of viral proteins [27]. Given the fact that quercetin also prevents progression of lung disease in COPD and modulates innate immunity of airway epithelial cells [14,28], quercetin treatment may be useful in treating RV-induced exacerbations in COPD.

## Conclusion

Although COPD patients do not show increased frequency of RV infections, they experience more severe and prolonged respiratory symptoms. Following RV infection, COPD patients also acquire secondary bacterial infections leading to progression of lung disease. This may be due to defective innate immune mechanisms of COPD airway epithelial cells that line the conductive airways. Therefore, strategies that repairs the airway epithelium, improve innate immune mechanisms may reduce the impact of RV-induced severe respiratory symptoms and progression of lung disease in this patient population. Quercetin, which has the capacity to modulate airway innate immune functions and inhibit viral replication may be used for treating COPD exacerbations. Preliminary clinical trial indicate that quercetin is safely-tolerated up to 2000 mg/day in COPD patients with mild to moderate lung disease (Han, Martinez and Sajjan, Unpublished results). However, further studies are required to establish the safety and efficacy of using quercetin as a therapy to treat COPD exacerbations.
